# Housekeepers’ Department

**Published:** 1869-10

**Authors:** 


### Housekeepers' Department.

BY A HOUSKEEPER.

### DISHES FOR THE SICK.

Containing useful hints, and simple, well tried receipts, especially adapted for the use of young house-keepers with limited incomes.

Care has been taken to make the receipts plain to persons ignorant of cookery, so that a lady may prepare any of the dishes without assistance. Cooking for the sick will also receive attention. A good nurse is frequently as desirable as a skillful physician, and food for the sick is often of the first importance. We have carefully gathered together many strengthening prescriptions which we hope may be of use to our invalid readers.

**Chicken Broth.**

Take an old fowl; stew it to pieces, with a little onion, if liked. Season highly with pepper and salt; skim and strain it.

**Arrowroot.**

Take care to get the best arrowroot, as many imitations are sold. Mix a dessertspoonful with a little cold water till it is smooth. Boil half a pint of milk; pour it on the arrowroot while boiling, stirring it all the time. Add a lump or two of sugar and a little lemon peel.

**Toast and Water.**

Toast stale, light bread. Immerse it, while hot, in a pitcher or tumbler of cold water; cover it for half an hour before drinking. Make it fresh once a day. The vessel may be refilled as the water is used.

**Mutton Broth.**

Take half a pound of mutton, remove the skin and fat; put it in a stew pan and cover with cold water; a pint to a half pound will be a good proportion. Let it simmer slowly an hour, then strain it. Season to taste.

**Birds for Convalescents.**

Lay the bird upon the gridiron; broil until a light brown color; then put it in a stew pan; pour over hot water enough to cover. Let it stew until tender. Season with butter, pepper and salt. Young chickens, nicely broiled and seasoned, form a tempting, and always a palatable dish for invalids.

**Port Wine Jelly.**

One pint of port wine; one ounce of isinglass; one ounce of sugar; a quarter of a pint of water. Put the isinglass and sugar into the water; set it over the fire till the isinglass is dissolved; then add the wine. Strain into a jar or mould, and let it set. Give a piece, about the size of a walnut, two or three times a day, to the patient.

### RECEIPES FOR WELL FOLKS.

**Muffins.**

One quart of milk, three eggs beat in separately and very light, one-half teaspooful of salt, flour enough to make it a little thicker than ordinary batter.

**Green Corn Cakes.**

One pint grated corn, three tablespoonsful sweet cream, one tablespoonful melted butter, one egg, one cup flour, a little salt. Mix and bake as pancakes.

**To Warm Cold Beef.**

Cut the meat in long and narrow slices of an inch thickness, leaving a little of the firm fat upon each. Season with salt, pepper, and mixed spices, dredge them with flour, and heat them (without any way of approaching to frying) in the gravy saved from the cold joint. Season with a shrewd onion, and a little vinegar. Garnish with sippets, and serve.

**A Rich Pudding.**

Line your dish with rich puff-paste, cover the bottom with preserved stoned cherries or any kind of dried fruit (or, as a substitute, Sultana raisins), with the grated rind and juice of a lemon; cover it with slices of roll buttered and cut thin, two or three more layers of preserve and roll to nearly the top of the dish, sifting the sugar between each layer: just before baking pour over it seven eggs well beaten, a spoonful of cream and plenty of brandy.

**Vegetable Soup.**

To a quarter of a pound fresh butter, boiling hot, add two onions chopped fine; let them stew. When they are soft, add two heads of celery, teacup each of corn, beans, cabbage, and tomatoes. Stir well with the butter and onions. Have ready a kettle of boiling water; pour over the vegetables a pint at a time, until as much as is needed is added. Boil until the vegetables are done. Salt and pepper to taste. Lay slices of toast at the bottom of the tureen, and pour on the soup.

**Ginger Pound Cake.**

Three-quarters of a pound of butter, three-quarters of a pound of sugar, six eggs, one pound and a half of flour, one pint of molasses, the grated rind of two large oranges, three tablespoonsful of ginger, two tablespoonsful of cinnamon, one tablespoonful of dissolved salæratus, or one large teaspoonful of dissolved carbonate of ammonia. Beat the Butter and sugar to a cream. Beat the eggs very light and add to it, then stir in all the other ingredients except the salæratus or ammonia. Beat the mixture very hard for several minutes, then stir in the salæratus or ammonia. Butter an earthern cake-mould or thick iron-pan, pour in the mixture and bake it in a moderate oven. If you bake it in an iron pan, line the pan with several thicknesses of stout paper well buttered.

---

**Turtle Soup.**

Boil the turtle very tender, remove all the bones, cut the meat into small pieces; season with a tablespoonful each of sweet-marjoram, sweet-basil, thyme and parseley; pepper and salt to taste; one nutmeg grated fine; a dozen cloves, the same of allspice. Tie these in a thin muslin and remove before sending to table; stir a large tablespoonful of browned flour into a quarter of a pound of butter; add this to the soup; pour over five quarts of boiling water and reduce by boiling to three quarts, boil gently. A quarter of an hour before it is done add the green fat, half a pint of wine, a lemon sliced thin, and forcemeat balls; simmering five minutes, take out the lemon peel. This is for a small turtle; if not fat, a slice of ham may be added, and remove before serving.

---

### A Precocious Child.

The St. Charles, (Minn.,) *Herald*, recently contained the following very singular account of a precocious child, reported by Dr. J. H. SUDDUTH, who is a respectable physician of St. Charles.

Much of the singularity of the case however, is absorbed in the fact that the child is a female. The doctor says:

"I was called upon a few days ago to attend a sick child, a daughter of William and Mary Jane Hearsey, living in the southwest part of St. Charles township. I found the child, aged a few days under five months, very ill. After administering medicine to the child, I was startled to hear it say very distinctly, "mamma, baby don't want any more. Completely nonplussed, I inquired of the mother how long the babe had talked. As though it was no unusual occurence, she cooly said it commenced talking a few days before it was three months old! deeply impressed with this unheard of and premature developement, I watched the child with the deepest interest. It does not prattle, as is usual with infants when first trying to talk, stumbling upon and straining at words. It speaks clearly and coherently, a regular sentance that clearly expresses its thoughts and ideas. It seems to think, and then expresses its thoughts calmly and clearly. It seems to note the anxiety and wishes of others. A little four year old brother was out of the house and several of the family inquired where he was. He soon came in, when the baby seeing him, said to his mother: "Otty has come home." It will lie quiety in its cradle while its mother is at work, and when it is hungry will say "baby wants dinner," or "mamma take baby up," as plainly as a child five or six years of age. I may as well mention the fact that another of the children commenced talking at eightmonths old."

---

BLEACHING SPONGES.—The white, beautiful appearing sponges which are sold in the streets of our cities, are bleached in the following manner: The softest, finest specimens are selected, and the sand removed from the cavities by shaking; they are then washed in hot water, and, after squeezing out the water, are placed in a bath of dilute hydrochloric (muriatic) acid, and allowed to remain for half an hour. They are then taken out, and, after washing again in hot water, are placed in a fresh bath of the dilute acid, to which has been added six per cent. of dissolved hyposulphite of soda, and allowed to remain twenty-four hours. The sponge is finished by washing in water, and drying.—*Boston Jour. Chem.*

---

—A newspaper is published on board the steamer Richmond, one of the huge passenger palaces which plys between Louisville and New Orleans. It is called the Richmond *Head Light.* It has two compositors and one editor, and is published six times during each trip, or about three times a week.

—A man was recently bitten by a rattlesnake in Greenfield, Iowa. Whisky killed the effects of the poison. It's hard finding anything that whisky, now-a-days, *won't* kill.

